# Conjonctival melanoma metastatic to the breast: a case report

**DOI:** 10.1186/1756-0500-7-621

**Published:** 2014-09-09

**Authors:** Saoussane Kharmoum, Mesmoudi Mohamed, Hafida Benhammane, Jinane kharmoum, Salima Touri, Khadija Setti, Youssef Bensouda, Saber Boutayeb, Ibrahim Elghissassi, Hind Mrabti, Bassma El Khannoussi, Yassir Sbitti, Hassan Errihani

**Affiliations:** Departement of Medical Oncology, National Institute of Oncology, Poste Madinat al Irfane, Rabat, BP 6213, Morocco; Departement of Medical Oncology, University Hospital Hassan II, Fes, Morocco; Departement of Pathology, Rabat, Morocco

**Keywords:** Conjunctival melanoma, Breast metastases, Metastatic melanoma

## Abstract

**Background:**

Breast metastasis is fairly uncommon and prognosis is dismal. Breast metastasis might be the first symptom or may occur during the course of other malignancies dominantly arising from the contralateral breast. Leukemia, lung cancer and conjunctival melanoma may spread to the breast.

**Case presentation:**

A 43-year-old female patient was operated on for conjunctival melanoma. After two years the disease progressed quickly and cutaneous nodes appeared on the back and paraumbilical region. Physical and radiological examination showed a breast mass. No palpable lymph’s nodes were noted. She underwent an open biopsy. Histopathologic examination and immunohistochemistry confirmed breast metastases from melanoma. During post-operative staging multiple nasopharyngeal and oropharyngeal lesions were also objective. The patient was given palliative dacarbazine (250 mg/m^2^ per day for 4 days) for 4 cycles. She died 4 months after the diagnosis of breast metastases.

**Conclusion:**

Histopathological evaluation should be mandatory in patients with medical history of malignancies in order to differentiate new primary tumors, metastases, and benign tumors.

## Background

Melanoma of the conjunctiva is a relatively rare and highly aggressive ocular malignancy [1]. These tumors’ may invade the orbit and the eye and metastasize to regional lymph nodes and systemically [2]. Breast metastases from extra mammary cancers are rare and melanoma is one of the malignancies that can metastasize to the breast. Benign and primary malignant breast tumors are quite common, but secondary tumors in the breast from metastatic malignancies are rare. Here we report a case of a young woman diagnosed with breast metastases from a conjunctival melanoma.

## Case presentation

**Figure 1:**
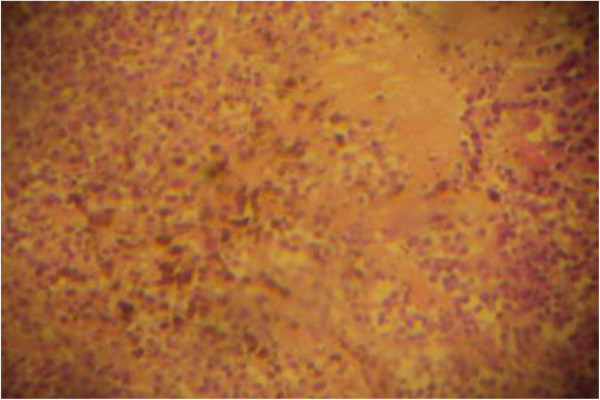
**Low power view showing a diffuse tumoral proliferation of round cells (Hematoxylin and eosin × 10).**

**Figure 2:**
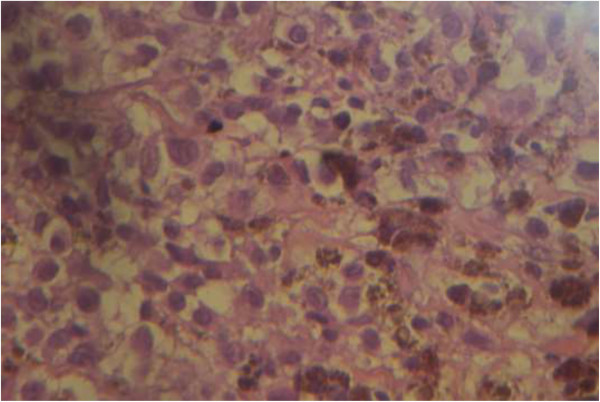
**Higher power view showing melanoma cells some of which are producing brown melanin pigment (Hematoxylin and eosin × 40).**

**Figure 3:**
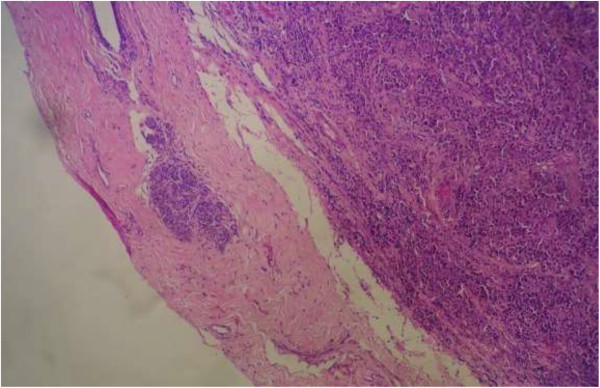
**Histologic section show a breast parenchyma infiltrated by melanocytic prolifération (hematoxylin and eosin × 10).**

**Figure 4:**
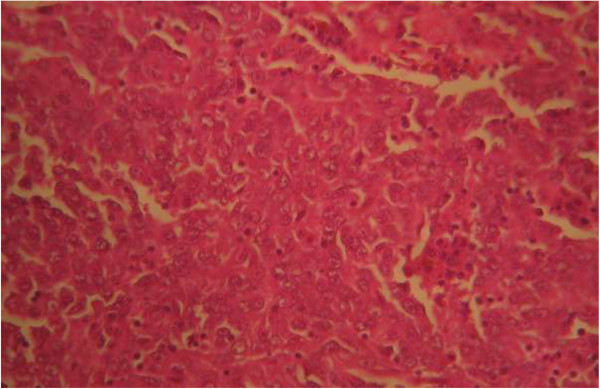
**Histopathologic section show a diffuse tumoral proliferation of round cells with large central nucleoli.** (Hematoxylin and eosin × 10).

**Figure 5:**
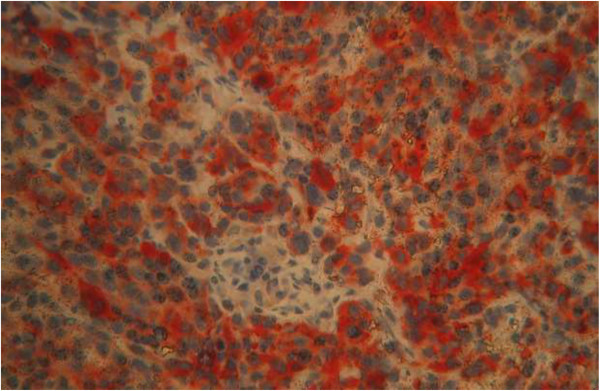
**Positive immunostaining of tumoral cells for HMB45 (magnification × 20).**

A 43-year-old female with no personal or familial pathological disease previously, and negative history of systemic disease treated by enucleation two years ago for primary conjunctival melanoma (Figures 1 and 2), without adjuvant radiotherapy. During regular follow-up visits no residual or recurrent lesion occurred. Two years after, she presented multiples cutaneous lesions. Physical examination demonstrates multiple cutaneous nodules on the back and paraumbilical region. Breast examination revealed a 2 cm hard lump in the left breast, and no palpable lymph nodes. Physical examination of the contralateral breast was normal. Mammography and ultrasound showed a lobulated contoured left breast mass reported as BI-RADS 4 (Breast Imaging-Reporting and Data System). An open biopsy was performed. Morphological examination showed a solid tumor suggesting a melanoma involvement (Figures 3 and 4). Immunoassaying was performed showing a negative staining for cytokeratin markers and hormonal receptors. However it showed strong positivity for the melanoma marker S-100 protein, and patchy staining for Melan-A and HMB-45 (Human Melanoma Black) (Figure 5). These findings concluded a diagnosis of breast metastatic disease from melanoma, and eliminate malignant or benign primary breast tumor. During post-operative staging with a whole body computed tomography (CT) Scan, multiple nasopharyngeal and the oropharyngeal metastases were also noted. Histopathological examination of these lesions confirmed features of metastatic disseminated disease from melanoma. She was given palliative dacarbazine (250 mg/m^2^ per day for 4 days). The patient received 4 cycles. She died 4 months later.

## Discussion

Invasive conjunctival melanoma accounts for only 1–2% of all ocular melanomas [1]. Similar to cutaneous melanomas, conjunctival melanomas originate from melanocytes that are derived from the neural crest. It is a potentially lethal neoplasm with an average 10-year mortality rate of 30% [2]. Systemic metastases occur in 14% to 27% of cases. Breast involvement in melanoma is not an isolated finding; it is usually associated with disseminated disease. Subcutaneous tissue, lung, liver, and brain are common secondary involvements in this disease [2]. Breast metastasis might be the first symptom or may occur during the course of other malignancies. Shetty et al [3], reported a review of literature, presenting data from 1855 to 1992, and found 431 cases of secondary extra-mammary breast tumors. The majority of them represented metastases from malignant melanoma (79 cases), followed by lung cancer metastases (78 cases), ovarian cancer (50 cases), and prostate (39 cases), kidney (24 cases), and other (143 cases). The time between diagnosis of the primary melanoma and the occurrence of a breast metastasis ranged from 13 to 180 months (median 62). Clinically, a breast lesion may mimic primary breast carcinoma. It’s necessary to differentiate primary to secondary extra-mammary tumors because prognosis and treatment of these neoplasm differ. Diagnosis of breast disease involves the work of multidisciplinary team of specialists. Radiologists perform necessary imaging for establishing optimal diagnosis. Core biopsy was done to obtain histological diagnosis. Immunocytochemical panel should be used to confirm the diagnosis of secondary metastatic melanoma to the breast. Our diagnosis was suspected by the comparative examination of the primary and metastases’ histological findings, and confirmed by a complete immunochemistry panel (negative staining for cytokeratin and positive for melan A and HMB45). Breast metastases are poor prognostic sign [4]. Radvel et al, reported a 12.9 months median time of survival after diagnosis of breast metastases [5]. In our case, the patient died 4 months after starting a treatment based on dacarbazine.

A recently published randomized controlled trial has shown that inhibitor (BRAF and MEK) kinase improved rates of overall and progression-free survival in patients with previously untreated melanoma with the BRAF V600E mutation [6].

## Conclusion

Metastasis to the breast must be considered in any patient with a known primary malignant tumor history who presents with a breast lump. Histopathological evaluation should be mandatory in patients with medical history of malignancies in order to differentiate new primary tumors, metastases, and benign tumors. Oncologists, surgeons, pathologists, and radiologists have to work together to reach the best possible therapy against this aggressive type of cancer.

## Consent

The initial consent was orally obtained from the patient, and the written informed consent was obtained from the patient's next of kin for publication of this case report and any accompanying images. A copy of the written consent is available for review by the Editor-in-Chief of this journal.
